# Characterizing HIV Acquisition Risk, Treatment Gaps, and Populations Reached Through Venue-Based Outreach and Clinical Services in Blantyre, Malawi: Findings From a District-wide CLOVE Study

**DOI:** 10.1097/QAI.0000000000003493

**Published:** 2024-07-18

**Authors:** Emmanuel Singogo, Sharon S. Weir, Evaristar Kudowa, Maganizo Chagomerana, John Chapola, Jessie K. Edwards, Confidence Banda, Gift Kawalazira, Yohane Kamgwira, Andreas Jahn, Sarah Bourdin, Thomas Hartney, Lucy Platt, Brian Rice, James R. Hargreaves, Mina C. Hosseinipour

**Affiliations:** a University of North Carolina Project, Lilongwe, Malawi;; b University of North Carolina, Chapel Hill, North Carolina;; c Blantyre District Health Office, Ministry of Health, Lilongwe, Malawi;; d Department of HIV &AIDs, Ministry of Health, Lilongwe, Malawi;; e London School of Hygiene and Tropical Medicine, London, UK; and; f School of Medicine and Population Health, University of Sheffield, Sheffield, England.

**Keywords:** HIV, social venues, suppression, priority populations

## Abstract

**Background:**

In 2017, Blantyre district had the highest adult HIV prevalence in Malawi (17.7%) and lowest viral suppression (60%). In response, the Ministry of Health expanded prevention and treatment services. We assessed whether outreach to social venues could identify individuals with increased HIV acquisition risk or with unsuppressed HIV not currently reached by clinic-based services.

**Methods:**

We conducted a cross-sectional biobehavioral survey in Blantyre, Malawi, from January to March 2022. We visited social venues where people meet new sexual partners and government clinics providing HIV testing or STI screening. Participants older than 15 years were interviewed and tested for HIV infection if not on ART. HIV recency tests were performed on those testing positive, and dried blood spots (DBS) were collected to quantify viral load and also to identify acute infection in those with HIV- results.

**Results:**

HIV prevalence (18.5% vs 8.3%) and unsuppressed HIV infection (3.9% vs 1.7%) were higher among venue-recruited (n = 1802) than among clinic-recruited participants (n = 2313). Among PLHIV at both clinics (n = 199) and venues (n = 289), 79% were virally suppressed. Few had acute (n = 1) or recent infection (n = 8). Among women, HIV prevalence was 4 times higher (38.9% venue vs 8.9% clinic). At clinics, PLHIV reporting visiting venues were less likely to be suppressed (54.6 vs 82.6%). More men at venues than at clinics reported paying for sex (49% vs 30%) or having multiple sex partners in the past 4 weeks (32% vs 16%).

**Conclusions:**

Enhanced venue-based prevention and testing for men and women could reduce treatment lapses, onward transmission, and improve HIV treatment outcomes.

## Abstract 2

Supplemental Digital Content is Available in the Text.

## INTRODUCTION

Despite an estimated 38% decline in new HIV infections since 2010, UNAIDS estimates that 1.3 million individuals acquired HIV in 2022. Approximately 71% of people living with HIV in 2022 had suppressed viral loads, but fewer men had suppressed viral load than women (67% for men vs 76% for women).^1^

Public health programs often lack evidence to tailor testing, treatment, and prevention services. Service providers may not know how many people living with HIV in their catchment area are not aware of their infection, linked to care, or virally suppressed. Although high viral loads during the brief period of acute infection increase the probability of onward transmission^2^ and affect epidemic trends,^3,4^ available cost-effective strategies to routinely detect acute HIV infection^5^ are rarely deployed.

In 2016, in response to the high adult HIV prevalence in Blantyre City (17.7%) and a low viral suppression rate (60%), the Ministry of Health expanded prevention and treatment services. Provider-initiated testing and counseling^6^ increased the uptake of testing at clinics, but clinic-based diagnosis may still miss people with unsuppressed HIV infection and people who need prevention services. Barriers to access may include failure of providers to initiate testing,^7^ lack of transportation,^8^ lack of interest in or fear of testing, long wait times,^9^ stigma-related barriers including fear of disclosure,^10^ and inaccuracy of perceived risk of infection.^11^

Venue-based outreach strategies are assumed to complement clinic-based strategies by reaching people missed by clinic-based services. The Priorities for Local AIDS Control Efforts (PLACE) method empirically identifies social venues where key members of sexual transmission networks, including mobile, stigmatized, and hard-to-reach populations, can be reached.^12–16^ Multiple PLACE studies in Malawi have found that people with high rates of new partner acquisition and inconsistent condom use^12,17^ could be reached at venues, but there is limited understanding of the extent to which venue-based methods identify people not reached by clinics.

In this study, we aimed to provide epidemiologic evidence to inform the district-wide HIV prevention response in Blantyre. We assessed whether an enhanced PLACE protocol could identify populations unreached with prevention and treatment services. We compared HIV outcomes and access to prevention and treatment services among venue attendees and clinic attendees.

## METHODS

### Study Design

We compared HIV status and sociodemographic characteristics of cross-sectional probability samples of people at social venues and patients at government clinics that provided HIV testing or STI screening in addition to other services offered at the clinic. To assess the overlap between populations and estimate the proportion of people at venues not reached by clinic services, we asked patients at clinics how frequently they visit social venues and people at venues how frequently they visit clinics. Clinic in this case is defined as a government health facility offering primary health care services including STI screening, HIV counseling and testing, and HIV Continuum of Care.

### Study Setting

Blantyre District's urban population is 918,000 and rural population is 531,000.^18^ Adult HIV prevalence declined from 17.7% in 2017^19^ to 14.2% in 2020^20^ to 13.7% in 2022. Adult prevalence remains higher in urban (15.2%) than in rural (10.9%) Blantyre in 2022. Our study was conducted in collaboration with the Ministry of Health, the National AIDS Control Program, and the Blantyre Prevention Strategy, a 5-year project to strengthen HIV prevention.

### Selection of Clinics

All government clinics that screened patients for STI and conducted at least 30 HIV tests per week were eligible. We randomly sampled 13 of the 21 eligible clinics, including 8 rural and 5 urban clinics (see Figure 2, Supplemental Digital Content, http://links.lww.com/QAI/C332).

### Selection of Venues

Identification of social venues and recruitment of participants followed the Priorities for Local AIDS Control Efforts (PLACE) method.^21^ First, 37 geographic areas likely to contain clusters of venues, dubbed “Priority Prevention Areas” (PPAs), were identified in consultation with stakeholders and validated by drive-throughs of the district. PPAs included trading centers, construction areas, tea estates, areas with nightlife and alcohol consumption, and areas known for sex work.

### Recruitment of Respondents at Selected Clinics and Venues

At clinics, eligible participants were patients seeking care. At venues, eligible participants were venue patrons or workers. The age of respondents was 15 years and older. Minors younger than 18 years were not eligible if they were accompanied by a parent or on a family errand. Participants were recruited using a take-all sampling approach. At clinics, this approach resulted in testing patients otherwise not eligible for testing due to clinic screening guidelines or provider decision.

Respondents were sequentially approached, invited to participate, and screened for eligibility. Those who consented were tested for HIV if they were not on ART.

### Data Collection

Participants were interviewed face-to-face using a preprogrammed structured questionnaire loaded onto an android tablet. Repeat visits to clinics and venues were made, if necessary, to meet survey targets.

Within the selected PPAs, interviewers asked a diverse group of 400 community informants (age 18 years and older who provided verbal informed consent) to identify venues, defined as physical buildings, outdoor sites, public events, or social media sites, where people go to meet new sexual partners. Named venues included nightclubs, hotels, lodges, truck stops, market days, and outdoor areas. Community informant interviews continued until no new venues were named.

Venues were validated during the busiest time of year through in-person visits to the venues in November and December 2021. Interviewers obtained GPS coordinates and interviewed a knowledgeable adult (such as a bar manager) about patron characteristics and on-site prevention services. After excluding duplicate, permanently closed, and venues not at the provided address, the sampling frame of venues included 709 verified locations. Of these, 161 locations were randomly sampled for the biobehavioral survey. Rural venues were oversampled (31%) compared with urban venues (21%).^8^

We conducted the biobehavioral survey concurrently in clinics and venues between January and March 2022. Forty-three venues were inaccessible or closed due to heavy rains or COVID-19–related factors (see Figure 3, Supplemental Digital Content, http://links.lww.com/QAI/C332). The interviewers collected information on participants' demographic characteristics, social vulnerabilities, sexual behavior, venue visiting behavior, clinic attendance, and access to HIV prevention, testing, and treatment services. Rapid HIV testing was conducted on-site by government-trained HIV testers, and testing result for each participant who consented and participated in biobehavioral survey was recorded in the tablet.

### Primary Outcome: HIV Status (Acute, Recent, Suppressed, Uninfected)

HIV testing was conducted for only participants who consented and were not on ART. From those with already HIV+ diagnosis, DBS samples were collected to monitor their viral suppression.

HIV testing was conducted by HIV Diagnostic Assistants trained by the Ministry of Health following nationally established protocols for rapid HIV testing, recency testing, and collection of DBS. Participants were first tested with the Alere Determine HIV-1/2 Ag/Ab Combo test. Those with a positive test received the Trinity Biotech Uni-Gold Recombigen HIV-1/2 test for confirmation. Those with a positive Uni-Gold test were tested for recent infection using the Asanté HIV-1 Rapid Recency Assay, asked to provide DBS for assessment of viral load, referred for treatment, and provided with standard referral slips to give to sexual partners/family members for testing. A “false” recent infection was defined as a “recent” Asante test result in the presence of HIV viral suppression (<1000 copies of HIV per ml).

Anyone testing HIV-negative provided DBS samples for assessment of acute infection. Assessment of acute infection was done by laboratory algorithms to detect HIV-RNA from pooled DBS samples at the Queen Elizabeth Central Hospital Laboratory in Blantyre. Confirmation of acute infection was done by the UNC Laboratory in Lilongwe.

**FIGURE 1.**
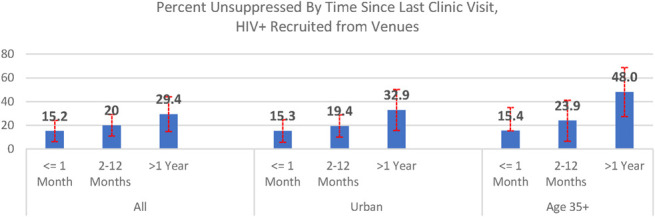
Percent unsuppressed by time since last clinic visit.

The primary HIV outcome was a 5-level variable (see Figure 1, Supplemental Digital Content, http://links.lww.com/QAI/C332) defined as:

1. HIV-Negative: required a negative rapid test and no detectable HIV RNA;
2. Acute Infection: required detectable HIV RNA in the absence of anti-HIV antibodies and viral load >5000 copies of HIV per ml of blood;
3. Recent Infection: required a confirmed HIV+ rapid test, a “recent” Asanté test result, and viral load ≥1000 copies of HIV per ml of blood;
4. Unsuppressed Long-Term infection: required confirmed HIV+ rapid test, a “long-term” infection per Asanté, and ≥1000 copies of HIV per ml of blood;
5. Suppressed Infection: required confirmed HIV+ rapid test and <1000 copies of HIV per ml of blood, including people with a “recent” infection identified by the Asante test but subsequently found to have viral loads <1000 copies of HIV per ml of blood.

### Inclusion/Exclusion Criteria

Of the total of 2459 clinic and 2000 venue potential participants approached, this analysis is limited to 2313 of 2453 eligible clinic patients (94%) and 1802 of 1988 eligible venue patrons and workers (91%). These participants provided informed consent and had complete testing and interview data (see Figure 4, Supplemental Digital Content, http://links.lww.com/QAI/C332). The most common reason for exclusion was lack of a viral load test to assess acute infection among those with an initial HIV-negative test result.

### Statistical Methods

We calculated survey weights based on a 2-stage sampling design, specifically as the inverse of the product of the probability that the venue or clinic was sampled and the probability that the respondent was sampled from the people within the venue or clinic. The design was take-all sampling within a venue or clinic. Each interviewer counted all men and women at each clinic or venue before each survey. We used these counts to adjust the sampling weights in situations where more people were counted than interviewed. All parameters were estimated using SAS version 9.4 (SAS Institute Inc.) survey procedures (with urban–rural strata and either clinic or venue as the cluster) and weights as described above.

We compared characteristics of participants by recruitment location (clinics or venues) and by sex and age. We compared the prevalence of HIV and unsuppressed HIV among venue and clinic populations using prevalence ratios. Then, 95% confidence intervals for point estimates and prevalence ratios were estimated with proc surveyfreq in SAS version 9.4 (SAS Institute Inc). The method computes variance estimates using Taylor series linearization and considers the multistage survey design with stratification (2 strata), clustering (131 clusters), and unequal weighting.

We assessed the association between HIV suppression and clinic attendance among the venue population by using the Cochran–Armitage test for trend available in SAS. Length of time since clinic visit was categorized as recent (4 weeks), moderate (less than a year), and long-term (over a year ago).

We defined testing yield as the number needed to test to find an unsuppressed case of HIV and estimated as the inverse of the prevalence of unsuppressed infection in the subgroup. To identify geographic clusters of unsuppressed infections at venues, we used sampling weights to estimate the number of cases at venues in each PPA.

### Ethical Review

The National Health Sciences Research Council in Malawi (Protocol #: 20/12/2637), the University of North Carolina in Chapel Hill, NC (Protocol #: 22018), and the London School of Hygiene and Tropical Medicine (ethics reference 22959) approved the protocol.

## RESULTS

### Comparison of Clinic and Venue Populations

#### Demographic Characteristics and Underlying Risk

A total of 2313 clinic patients and 1802 social venue patrons and workers were included in the analysis. Venue participants were more likely than clinic participants to be male (69% vs 25%), aged 25–50 years (62% vs 52%), unmarried (62% vs 40%), previously arrested (28% vs 8%), and daily consumers of alcohol (44% vs 7%). In fact, 22% of all venue participants (compared with 3% of clinic participants) were male, aged 25+ years, and reported drinking alcohol daily (data not shown). Clinic women were more likely to be pregnant than women from venues (8% vs 1%) (Table 1).

**TABLE 1.** Characteristics of Clinic and Social Venue Participants

Item Description	Clinic			Venue		
	Unweighted N	Weighted %	95% CI	Unweighted N	Weighted %	95% CI
Complete interview	2313	100.0		1802	100.0	
Demographic characteristics						
Male	749	25.3	18.3 to 32.3	1368	68.9	65.3 to 72.6
Female	1564	74.7	67.7 to 81.7	434	31.1	27.4 to 34.7
Age group						
15–24	979	44.6	38.4 to 50.9	597	34.8	30.4 to 39.2
25–50	1254	52.0	47.4 to 56.6	1155	62.3	58.3 to 66.4
>50	80	3.4	1.2 to 5.5	50	2.8	1 to 4.7
Married or living with partner	1371	60.4	54.9 to 65.9	740	37.7	33.9 to 41.6
Currently pregnant (women only)	124	8.3	5.7 to 10.8	5	0.6	0 to 1.2
Rural residence	1568	75.8	64.8 to 86.8	1179	62.4	54.5 to 70.0
Underlying determinants of risk						
Did not complete secondary school	1612	71.5	67.4 to 75.6	1232	68.2	64.3 to 72
Not enough food past 12 mo	1011	44.8	36.4 to 53.2	697	37.3	33.5 to 41
Ever arrested	189	7.8	6 to 9.5	516	27.5	24.2 to 30.8
Drinks alcohol daily	206	7.1	5.4 to 8.8	800	43.8	39.2 to 48.4
Venue and clinic visits						
Visited clinic in past year	2313	100.0		1202	68.5	65.0 to 72.0
Visited clinic in past 6 mo	2313	100.0		932	53.9	50.3 to 57.5
Visited venue and clinic in past 4 wk	332	12.4	9.4 to 15.4	335	20.4	17.8 to 22.9
Only visited clinic in past 4 wk	1981	87.6	84.6 to 90.6	0		
Only visited venue in past 4 wk	0	0.0		1467	79.6	77.1 to 82.2
Lives at venue or visits daily	—	—		1037	58.8	53.3 to 64.4
Use of health services						
Avoided health care because of stigma or discrimination	103	4.2	2.7 to 5.8	94	4.1	3.1 to 5.2
Rated recent service received at public clinic as good or excellent	2054	88.8	84.5 to 93.1	1566	86.7	83.8 to 89.6
Ever tested for COVID-19	568	21.9	18.8 to 25	390	21.1	18 to 24.3
Circumcised (men only)	506	67.2	58.9 to 75.5	880	62.8	58.2 to 67.5
In the past 12 mo:						
STI test or examination	367	17.0	12.8 to 21.3	290	15.6	13.5 to 17.7
Provided TB sputum sample	251	10.1	8 to 12.2	167	9.7	8 to 11.4
Received HIV information at this location	958	40.6	30.9 to 50.4	399	21.8	18.7 to 24.9
Reports easy to get a condom	1701	73.5	66.6 to 80.4	1451	81.7	79.2 to 84.2
Condoms free in past 6 mo	720	29.0	24.6 to 33.4	874	50.4	45.7 to 55.1
Showed condom to interviewer	80	3.0	1.4 to 4.7	238	16.4	12.2 to 20.6
HIV testing						
Knows where HIV test in Blantyre	2010	86.0	80.5 to 91.5	1538	85.0	81.9 to 88.1
Reports not more difficult to get HIV test during COVID-19 pandemic	2010	84.6	75.7 to 93.5	1534	84.7	81.5 to 88
Ever tested for HIV	2038	86.9	81.7 to 92	1459	82.5	79.6 to 85.4
Ever tested for HIV in Blantyre	1713	73.4	64.4 to 82.4	1170	63.5	59.7 to 67.4

N, population size.

#### Venue and Clinic Visiting Profiles

Clinic and venue populations are not mutually exclusive. Among the clinic population, 12% had visited a venue in the past 4 weeks. Among the venue population, 20% had visited a clinic in the past 4 weeks, 54% in the past 6 months, and 68% in the past year. A third (32%) had not visited a clinic in over a year. Among clinic attenders, men were more likely than women to have recently visited a venue (31% vs 6%). Among venue attenders, a higher percentage of women than men reported visiting a clinic in the past 4 weeks (29% vs 17%), 6 months (65% vs 49%), and 1 year (78% vs 65%). Approximately 27% of venue-based women reported living at the venue and 59% of all venue participants reported either living at the venue or visiting the venue daily.

#### Gaps in Access to and Use of Prevention Health Services

Self-reported STI, TB, HIV, and COVID-19 testing history did not differ significantly by clinic and venue groups. More clinic-based participants reported receiving HIV information on-site than the venue-based sample (41% vs 22%), but venue-based participants were more likely to have received free condoms in the past 6 months (50% vs 29%) and more likely to show a condom to the interviewer when asked (16% vs 3%). Overall, 87% of venue participants and 89% of clinic participants rated the most recent service received as a public clinic as good or excellent. Four percent reported avoiding health care in the past year due to stigma or discrimination from health workers.

#### Sexual Behavior

The venue-based population reported significantly more sexual partners in the past 7 days, 4 weeks, and 1 year than the clinic population (Table 2). Nearly 90% of venue-based women reported a new sexual partner in the past month. Venue-based women were 10 times more likely to report being paid for sex than clinic-based women (58.4% vs 4.6%). Half (48.8%) of the venue-based men and 29.9% of clinic-based men reported paying a woman for sex in the past year. In both populations, women were more likely to report anal sex than men. Fewer than 10% in any group reported meeting partners online or on a phone app. Among men, 31% at clinics and 21% at venues reported they had never used a condom.

**TABLE 2.** Self-Reported Sexual Behavior by Sex Among Clinic and Venue Participants

	Men					
	Clinic			Venue		
	Unweighted N	Weighted %	95% CI	Unweighted N	Weighted %	95% CI
Total	749	100		1368	100	
Sexually active in the past year	702	93.1	89.7 to 96.5	1282	94.3	92.5 to 96.0
2+ partners in the past 7 d	83	11.2	6.6 to 15.9	242	19.5	16.7 to 22.3
2+ partners in the past 4 wk	127	16.3	11.3 to 21.3	409	32.0	28.7 to 35.3
10+ partners in the past 12 mo	27	2.9	1.9 to 4	122	9.8	7.9 to 11.8
New partner in the past 4 wk	166	27.9	22.4 to 33.4	606	56.0	51.4 to 60.7
Met partner online/phone app	65	8.5	4.8 to 12.2	101	7.6	5.5 to 9.7
Anal sex with A man	3	0.3	0 to 0.6	10	0.8	0.3 to 1.4
Paid a woman for sex	221	29.9	24.8 to 35	658	48.8	44.8 to 52.9
Never used a condom	193	30.5	18.8 to 42.3	269	20.7	16.8 to 24.6
Used a condom during most recent vaginal sex	243	31.9	23.2 to 40.6	501	40.1	36 to 44.2
Used a condom every time past 6 mo	154	21.9	12.7 to 31	341	28.7	24.5 to 32.9

	Women					
	Unweighted N	Weighted %	95% CI	Unweighted N	Weighted %	95% CI
Total	1564	100		434	100	
Sexually active in the past year	1453	93.2	90.4 to 96.0	412	94.8	91.8 to 97.7
2+ partners in the past 7 d	67	4.8	3.7 to 5.9	208	48.9	36.6 to 61.3
2+ partners in the past 4 wk	100	7.0	4.5 to 9.5	239	59.9	47.6 to 72.3
10+ partners in the past 12 mo	14	0.9	0.1 to 1.7	176	45.8	32.6 to 59
New partner in the past 4 wk	1222	79.3	76.9 to 81.7	378	89.7	83.9 to 95.5
Met partner online/phone app	29	2.0	1.6 to 2.4	40	9.6	5.2 to 14.1
Anal sex with A man	27	2.2	0.8 to 3.7	21	6.5	1.6 to 11.4
Was paid for sex	74	4.6	3.1 to 6.1	234	58.4	46.9 to 69.9
Never used a condom	576	38.6	29.8 to 47.3	104	21.9	14.2 to 29.5
Used a condom during most recent vaginal sex	265	18.9	12 to 25.8	186	53.7	43.2 to 64.3
Used a condom every time past 6 mo	177	12.7	6.8 to 18.6	113	32.8	20.5 to 45.2

### Primary Outcome

HIV prevalence (18.5% vs 8.3%) and unsuppressed HIV infection (3.9% vs 1.7%) were higher among venue-recruited (n = 1802) than among clinic-recruited participants (n = 2313). The venue vs clinic difference in HIV prevalence was greater among women (venue: 38.8% vs clinic: 8.9%) than among men (venue: 9.4% vs clinic: 6.4%). There was 1 acute infection in the venue sample and none in the clinic sample. There were 6 recent infections in the venue sample and 2 in the clinic sample (Table 3). At venues, factors associated with HIV infection include female sex (39% vs 10%), reporting a new partner in the past 4 weeks (28% vs 13%), and reporting transactional sex (25% vs 13%).

**TABLE 3.** HIV Status and Primary HIV Outcome by Study Location and Sex of Participants

	Clinic			Venue		
	Unweighted N	Weighted %	95% CI	Unweighted N	Weighted %	95% CI
All						
HIV status						
HIV-negative	2114	91.7	90.1 to 93.3	1513	81.5	77.7 to 85.3
HIV-positive	199	8.3	6.7 to 9.9	289	18.5	14.7 to 22.3
Total	2313			1802		
Primary HIV outcome						
HIV-negative	2114	91.7	90.1 to 93.3	1513	81.5	77.7 to 85.3
Suppressed infection	152	6.6	5.4,7.7	224	14.6	11.0 to 18.2
Acute infection	0	0.0	—	1	0.0	0.0,0.1
Recent infection	2	0.1	0.0 to 0.2	6	0.4	0.6 to 0.7
Long-term unsuppressed	45	1.6	1.0 to 2.2	58	3.5	2.3 to 4.8
Total	2313			1802		
Urban participants						
HIV status						
HIV-negative	1209	90.3	88.2 to 92.3	1207	81.2	77.0 to 85.4
HIV-positive	118	9.7	7.7 to 11.8	238	18.7	14.6 to 23.0
Rural participants						
HIV status						
HIV-negative	905	92.9	90.8 to 94.9	306	83.3	77.1 to 89.5
HIV-positive	81	7.1	5.1 to 9.2	51	16.7	10.5 to 22.9
Men						
HIV status						
HIV-negative	699	93.6	91.3 to 95.9	1241	90.6	88.5 to 92.8
HIV-positive	50	6.4	4.1 to 8.7	127	9.4	7.2 to 11.5
Total	749			1368		
Primary HIV outcome						
HIV-negative	699	93.6	91.3 to 95.9	1241	90.6	88.5 to 92.8
Suppressed infection	32	4.6	2.8 to 6.3	84	5.6	4.2 to 7.0
Acute infection	0	0	—	0	0	—
Recent infection	0	0	—	3	0.3	0.0 to 0.6
Long-term unsuppressed	18	1.8	0.8 to 2.9	40	3.5	2.1 to 4.9
Total	749			1368		
Women						
HIV status						
HIV-negative	1415	91.1	89.2 to 92.3	272	61.2	51.9 to 70.4
HIV-positive	149	8.9	7.0 to 10.8	162	38.8	29.6 to 48.1
Total	1564			434		
Primary HIV outcome						
HIV-negative	1415	91.1	89.2 to 92.3	272	61.2	51.9 to 70.4
Suppressed infection	120	7.2	5.7 to 8.8	140	34.5	24.9 to 44.2
Acute infection	0	0.0	—	1	0.1	0.0 to 0.2
Recent infection	2	0.1	0.0 to 0.3	3	0.6	0.0 to 1.4
Long-term unsuppressed	27	1.5	1.0 to 2.1	18	3.6	1.6 to 5.6
Total	1564					

Although the overall percentage of the venue-based population that was unsuppressed (3.9% of 1802) was higher than the percentage of the clinic-based population that was unsuppressed (1.7% of 2313), when the comparison is limited to those who are HIV+, the proportion suppressed at both clinics (n = 199) and venues (n = 289) was similar (79%).

### Unsuppressed Infection

Among clinic participants, younger and married people were less likely to be suppressed. Among venue participants, older people and unmarried people were less likely to be suppressed. In both groups, men were less likely than women to be suppressed (see Table 1, Supplemental Digital Content, http://links.lww.com/QAI/C332). Within venues, older participants (aged 25+ years, PR: 2.1, 95% CI: 1.1 to 3.9) and those separated or divorced/widowed (PR: 2.6, 95% CI: 1.6 to 4.3) had increased risk of being unsuppressed. The same trend was observed among clinic attendees (see Table 1, Supplemental Digital Content, http://links.lww.com/QAI/C332). In addition, clinic participants who visited venues were less likely to be virally suppressed than other PLHIV clinic participants (53% vs 81%, not shown in the table).

#### Time Since Clinic Visit

Among PLHIV recruited from venues, time since clinic visit was associated with unsuppressed infection overall, among people recruited from urban venues, and among those aged 35+ years (test for trend *P* < 0.05) (Fig. 1).

#### Priority Prevention Areas

We estimated 907 unsuppressed infections among people attending venues at a busy time in Blantyre. These infections were geographically clustered in 4 Priority Prevention Areas in Blantyre (see Figure 5, Supplemental Digital Content, http://links.lww.com/QAI/C332).

#### Number Needed to Test to Find One Unsuppressed Infection

**FIGURE 2.**
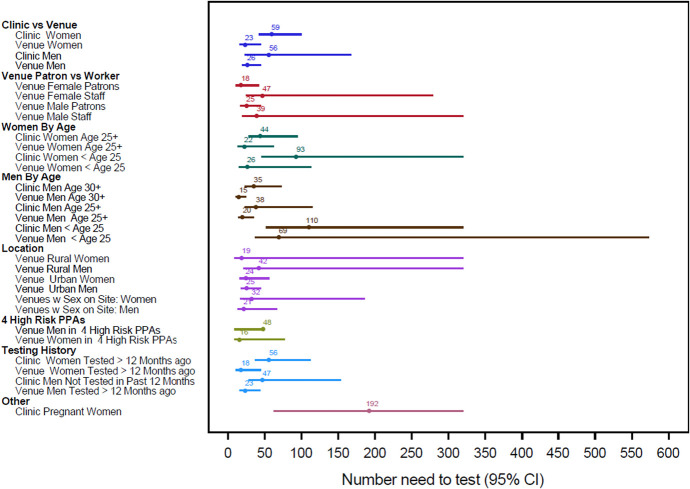
Number needed to test to find 1 unsuppressed infection categorized by sex, age, participant type, and study site.

The number of people needed to test at venues to find 1 case of unsuppressed HIV infection was lower than the number needed to test at clinics for similar categories (Fig. 2). Yield was highest among men aged 30 years and older at venues, where testing 15 would yield 1 unsuppressed infection, and lowest among pregnant women, where testing 200 would identify 1 unsuppressed infection.

## DISCUSSION

Our study found a higher prevalence of HIV (18.5% vs 8.3%) and unsuppressed infection (3.9% vs 1.7%) among people at social venues than among people attending health clinics. The venue-based population was more likely to be male (68% vs 28%) and report more sexual partners in the past 4 weeks and year. Among those with HIV infection, 79% of the clinic and venue populations had achieved viral suppression. Our detection of only 1 acute and 8 recent infections among the 4115 people tested at clinics or venues should be a welcome finding for HIV prevention and treatment efforts in Blantyre.

In terms of venue attendees, the majority were from the Blantyre district, with only approximately 1.5% of participants indicating they came from outside Blantyre. The majority of venue attendees were male but venue and clinic populations were not mutually exclusive. Among the venue population, close to 32% of them did not visit the clinic in the past year.

Historically, Blantyre has had the highest prevalence of HIV infection in the country with recent evidence of a decline. The most recent population-based household survey in Blantyre City, published after our data had been collected, found a reduction in HIV prevalence between 2016 and 2020 from 14% to 11.3% among men and from 21.8% to 17.1% among women.^19,20,22^ The most recent biobehavioral survey among female sex workers also found a reduction in the prevalence of HIV, from 62.7% in 2013 to 49.9% in 2020.^23^ The estimated point prevalence of HIV infection in our study among urban men (6.3% at clinics and 9.9% at venues) was lower than the prevalence reported in the MPHIA population-based study for Blantyre City (11.3%); however, our study population was younger than the MPHIA population and thus would be expected to have a lower HIV prevalence. Approximately 16% of men and women were older than 50 years in the MPHIA study compared with 5% in our study. Although women at urban venues represent a small percentage of the general population, they had a higher prevalence of infection than the general population of women from Blantyre City in the MPHIA study (38% vs 17%).

The proportion of PLHIV with suppressed viral loads in clinic and venue populations (79%) was similar to findings from the 2020 MPHIA study (81%)^23^ and consistent with other studies in the sub-Saharan region among female sex workers and men who have sex with men.^24^ Strategies to improve ART adherence among venue-based populations, which are often mobile, include differentiated care models such as individual client-centered models,^24^ and a multilevel comprehensive care package that includes mental health to improve adherence^25–27^

Given previous HIV prevalence estimates, we had expected more unsuppressed, recent, and acute infections in the venue population. Previous estimates of the prevalence of acute infection in Malawi^28^ led us to expect at least 10 acute infections in our sample, whereas we only detected one.

Similar to results from previous PLACE studies in Malawi, we identified subgroups with high rates of sexual partnerships and inconsistent condom use.^12,29^ HIV prevention programs targeting priority populations have greatly benefited from recent advances in biomedical HIV prevention interventions such as use of preexposure prophylaxis (PrEP) and differentiated HIV testing and outreach approaches.^25,30,31^ However, uptake, acceptability, and scalability of PrEP services are still ongoing, with high-risk populations being prioritized in estimating number of people who need PrEP.^32–34^

Male circumcision was surprisingly high among both the clinic and venue populations (67% in clinic vs 63% in venues). In 2016, the Malawi Demographic and Health Survey reported about 44% of men in Blantyre were circumcised.^35^ The high circumcision prevalence in our study could be due to positive impact of scale-up of voluntary medical male circumcision funded by PEPFAR, that designated Blantyre as a priority district, with men aged 15–29 years prioritized.

Limitations of the survey include conducting interviews during the COVID-19 pandemic and the rainy season when venue attendance was low. Although we determined HIV status, including acute HIV infection, and viral load assessment with laboratory tests, we did not test for evidence of antiretroviral therapy (ART). Our study confirms the finding in other studies in Malawi that people underreport HIV infection and taking ART.^19,20^ Although 5% of participants were missing viral load data, the missing seems to be at random.

The strengths of this survey include the inclusion of urban and rural areas of Blantyre, systematic and thorough venue identification and mapping, take-all sampling of individuals at venues and clinics, the use of highly experienced testing and counseling staff who had been trained by the Ministry of Health, the trained survey team, the assessment of acute HIV infection, and the assessment of viral loads for each participant. We believe we conducted as rigorous a search for pockets of undetected and unsuppressed HIV infection as is feasible in Blantyre.

## CONCLUSIONS

Although overall adult HIV prevalence was lower than expected, we found clear differences in HIV prevalence and behavioral risk between the mostly male venue-based population and the predominately female clinic population. Testing for acute or recent infection yielded few cases, leaving us to question its value for surveillance at the population level. The proportion of people living with HIV who had achieved viral suppression was similar in clinic and venues, but did not reach country's 95%-95%-95% targets. Efforts to address barriers to treatment access and biomedical interventions such as PrEP are still needed. Only half of the venue population had received free condoms in the past 6 months, suggesting venue-based outreach services are not adequately prioritized. Mapping of venues can improve provision of HIV services, hence outreach to priority prevention areas with higher prevalence of unsuppressed infection may advance prevention goals. Our study also shows that people who usually have high transmission potential are most often hidden, hence the need to improve or find alternative screening algorithms at clinics for identifying those most in need of prevention services. To learn how local HIV epidemics is changing in the district, HIV programs ought to use local data such as these cross-sectional surveys.

## Supplementary Material

SUPPLEMENTARY MATERIAL
