# Supplementary material for: Characterising HIV acquisition risk, treatment gaps and populations reached through venue-based outreach and clinical services in Blantyre, Malawi: findings from a district-wide CLOVE Study
Source: J Acquir Immune Defic Syndr. Author manuscript; Available in PMC 2025 Jan 15. (PMC11500693; doi:10.1097/QAI.0000000000003493)
Supplement: Supplemental Digital Content [file NIHMS2008837-supplement-Supplemental_Digital_Content.docx]

**SUPPLEMENTAL MATERIALS- METHODS**

**HIV testing outcomes**

The primary HIV outcome was a 5-level variable (Figure 1) defined as:

1. HIV Negative: required a negative rapid test and no detectable HIV RNA;
2. Acute Infection: required detectable HIV RNA in the absence of anti-HIV antibodies and viral load > 5000 copies of HIV per ml of blood;
3. Recent Infection: required a confirmed HIV+ rapid test, a “recent” Asanté™ test result, and viral load >=1000 copies of HIV per ml of blood;
4. Unsuppressed Long-Term infection: required confirmed HIV+ rapid test, a “long-term” infection per Asanté™, and >=1000 copies of HIV per ml of blood;
5. Suppressed Infection: required confirmed HIV+ rapid test and < 1000 copies of HIV per ml of blood, including people with a “recent” infection identified by the Asante test but subsequently found to have viral loads <1000 copies of HIV per ml of blood.

^1 Alere Determine HIV-1/2 Ag/Ab Combo test.2 Uni-Gold™ Recombigen® HIV-1/2 rapid test, 3 From DBS 4 From DBS 5^ [^Asanté ® HIV-1 Rapid Recency ® Assay^](https://www.sediabio.com/asante-hiv-1-rapid-recency/)

**Figure 1: HIV testing outcomes**

*Selection of Clinics and Recruitment of Patients*

The sampling frame was constructed based on routine HIV data from early 2021. At the time of the study, 46 of 100 health clinics in Blantyre conducted HIV testing and screening for STI. Of these, 21 government clinics conducted 30 or more HIV tests per week. Of these 21 eligible clinics, we randomly sampled 13 clinics (Figure 2).

Eligible participants for the clinic-based survey were patients aged 15 or older seeking care at Ministry of Health public clinics that provide HIV testing and screening for STI. Patients aged 15-17 were not eligible if they were accompanied by a parent. Informed consent was required of all participants.

Interviewers were allocated across clinics proportionate to the expected number of clients at a clinic. All patients presenting to selected clinics on survey days were sequentially approached, invited to participate, asked to undergo the informed consent process, and screened for eligibility. After consenting, participants were tested for HIV and interviewed face-to-face using a pre-programmed structured questionnaire loaded onto a tablet. Revisits to clinics were made to meet survey targets.

**Figure 2: Clinic selection process**

*Venue Identification, Selection and Recruitment of Patrons and Workers*

A sampling frame of venues was created by first identifying 37 neighborhoods dubbed “Priority Prevention Areas” (PPAs) in Blantyre District based on stakeholder input and validated by drive-throughs of the district with study team. PPAs were often commercial or trading centers, areas with high male to female ratio such as construction areas or tea estates, areas with nightlife and alcohol consumption, and/or areas known for sex work.

Subsequently, trained interviewers asked 400 community informants throughout the 37 PPAs to identify by name and address up to ten venues in the area. A venue was defined as a physical building, outdoor site, event or social media website where people go to meet new sexual partners. Examples include nightclubs, hotels, lodges, truck stops, market days and outdoor areas. In eliciting names of venues, interviewers probed community informants to name venues visited by sex workers, men who have sex with men and people who inject drugs, but attendance by key populations was not required.

Community informants, aged 18 and older, provided anonymous verbal informed consent and were purposively recruited based on targets to achieve a diverse profile of informants (e.g., bar owners, unemployed men, shop keepers, sex workers, transportation workers). Interviews continued until no new venues were named. After initial efforts to remove duplicate venues, we constructed an initial sampling frame with urban and rural strata comprised of 930 venues.

Next, interviewers attempted to visit all 930 named venues, obtain GPS coordinates of the venue, and interview a knowledgeable person aged 18 or older (such as a bar manager) about the characteristics of venue clientele, its busy times, onsite HIV prevention efforts and the type and number of key populations patronizing the venue. Venue informants provided verbal informed consent. 222 venues were removed from the sampling frame because they were permanently closed, not at the provided address or a duplicate of another venue. The remaining 708 venues (including 34 not visited due to heavy rains) were eligible for sampling for the bio-behavioral survey.

We selected a random sample of venues within each stratum with oversampling of the rural stratum. We oversampled rural venues because we expected that gaps in access to prevention and treatment services would be greater in rural areas. Some venues had closed between visits to the venue for venue characterization and visits to the venues to conduct the bio-behavioral survey. Venue mapping occurred during the holidays in December when venues were more popular. By February and March, many venues had closed, had fewer patrons due to the Covid-19 pandemic, or were less accessible due to heavy rains and poor roads. 28 of 119 randomly selected urban venues and 15 of 42 randomly selected rural venues were no longer accessible in February.

See Figure 3.

**Figure 3:** *Venue Sampling Design*

*Inclusion/exclusion criteria for this analysis*

This analysis is limited to 2313 clinic patients and 1802 venue patrons and workers who were eligible to participate, provided informed consent, and had complete testing data (Figure 4).

**Figure 4: Study Participation and inclusion in final analysis dataset**

**SUPPLEMENTARY MATERIAL- RESULTS**

**Unsuppressed Infection**

Among clinic participants, younger and married people were less likely to be suppressed. Among venue participants, older people and unmarried people were less likely to be suppressed. In both groups, men were less likely than women to be suppressed. (See Table 4). Clinic participants who visited venues were less likely to be virally suppressed than other PLHIV clinic participants (53% vs 81%).

**Table 1: Prevalence ratios (PR) and 95% confidence intervals (CI) for the total association between Participant Characteristics and Unsuppressed Long-term Infection**

|  | **Clinic (n=2313)** | | **Venue (n=1802)** | |
| --- | --- | --- | --- | --- |
|  | **PR** | **95% CI** | **PR** | **95% CI** |
| **Demographic Characteristics** |  |  |  |  |
| *Age 25+* | 4.0 | 2.1, 7.6 | 2.1 | 1.1, 3.9 |
| *Female* | 0.9 | 0.5, 1.8 | 1.1 | 0.6, 2.1 |
| *Separated/Widowed/Divorced* | 2.8 | 1.7, 4.6 | 2.6 | 1.6, 4.3 |
| *Urban* | 1.9 | 0.8, 4.3 | 1.2 | 0.6, 2.2 |
| **Underlying Determinants of Risk** |  |  |  |  |
| *Did not complete secondary school* | 1.3 | 0.5, 3.1 | 1.2 | 0.6, 2.3 |
| *Not enough food past 12 months* | 1.6 | 0.7, 3.3 | 1.4 | 0.8, 2.4 |
| *Ever arrested* | 2.3 | 0.7, 7.6 | 1.0 | 0.5, 2.2 |
| *Drinks Alcohol Daily* | 0.5 | 0.2, 1.1 | 1.1 | 0.6, 2 |
| *Visited venue in past 4 weeks* | 2.5 | 1.1, 5.5 | * |  |
| **Sexual Behavior** |  |  |  |  |
| *Paid or was paid for sex* | 2.2 | 0.9, 5.3 | 1.4 | 0.7, 2.8 |
| *New sex partner in past 4 weeks* | 1.2 | 0.4, 3.2 | 1.6 | 0.8, 3.2 |
| *10+ partners past year* | 1.3 | 0.2, 9.7 | 1.2 | 0.6, 2.4 |
| *Never used a condom* | 2.8 | 0.7, 10.2 | 0.8 | 0.4, 1.6 |
| **Health Services** |  |  |  |  |
| *Reports difficult to get condoms* | 1.4 | 0.4, 4.6 | 1.3 | 0.6, 2.6 |
| *Did not visit clinic in past year* | -- | -- | 1.3 | 0.7, 2.2 |
| *Reports health care stigma* | 1.4 | 0.3, 5.9 | 0.4 | 0.1,1.8 |
| *Among HIV+: Does not take ART* | ** | -- | 2.3 | 0.3, 18.0 |

^* Not applicable. Everyone visited venue. ** Not applicable. Everyone reported taking ART.^

*Priority Prevention Areas*

We estimated 907 unsuppressed infections among people attending venues at a busy time in Blantyre. These infections were geographically clustered in 4 Priority Prevention Areas in Blantyre (Figure 3).


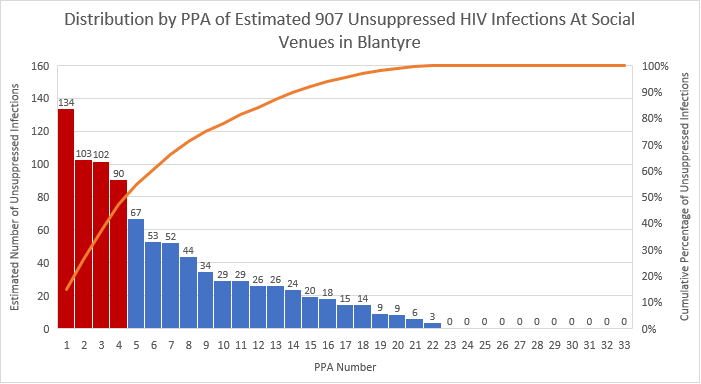


**Figure 5: Geographic Clustering of Infection**
